# Screen-Printed Sensors for Colorimetric Detection of Hydrogen Sulfide in Ambient Air

**DOI:** 10.3390/s19051182

**Published:** 2019-03-08

**Authors:** Laura Engel, Karina R. Tarantik, Carolin Pannek, Jürgen Wöllenstein

**Affiliations:** 1 Fraunhofer Institute for Physical Measurement Techniques IPM, 79110 Freiburg, Germany; karina.tarantik@ipm.fraunhofer.de (K.R.T.); carolin.pannek@ipm.fraunhofer.de (C.P.); juergen.woellenstein@ipm.fraunhofer.de (J.W.); 2 Department of Microsystems Engineering—IMTEK, University of Freiburg, 79110 Freiburg, Germany

**Keywords:** colorimetric paper sensor, hydrogen sulfide detection, Cu-PAN complex, screen-printing, UV/Vis spectroscopy, ambient air

## Abstract

A fast and sensitive method to monitor hydrogen sulfide (H_2_S) in ambient air based on a visible color change of a printed disposable sensor has been developed. As gas-sensitive material, an immobilized copper(II) complex of the azo dye 1-(2-pyridylazo)-2-naphtol (H-PAN) was synthesized and prepared in an ethyl cellulose matrix for screen printing. If H_2_S is present in ambient air, the gas sensitive layer changes its color from purple to yellow. A pre-primed polyethylene (PE) foil and a coated offset paper served as the printing substrate. The colorimetric response to the target gas was measured by UV/Vis spectroscopy in reflection at H_2_S concentrations between 1 to 20 ppm. Possible cross-sensitivities of the printed sensors towards methane (CH_4_), formaldehyde (CH_2_O), carbon monoxide (CO), ammonia (NH_3_), and nitrogen dioxide (NO_2_), as well as the long-term stability was investigated. Furthermore, reflection measurements of the Cu-PAN complex on an amorphous silica powder under gas admission served as preliminary test for the subsequent paste development.

## 1. Introduction

The detection of the toxic gas hydrogen sulfide (H_2_S), which is well known for its characteristic stench of rotten eggs, has major importance for the oil and gas industry, as well as other industries, such as wastewater treatment plants. These chemical industries produce H_2_S gas continually, although it is extremely hazardous for living beings. Even at low gas concentrations and short exposure time, H_2_S can cause substantial physical damage. Its toxicity is primarily due to the inhibition of metal-containing enzymes through sulfide formation or through the reduction of disulfide bridges [1]. For this reason, the particularly affected areas are the mucous membranes and tissues with a high oxygen demand, such as nerves and the heart [1]. Moreover, at higher gas concentrations (>100 ppm), H_2_S has the treacherous ability to stun the olfactory receptors [2], and the human nose can no longer be used as a reliable detector. All of this shows that the effect of H_2_S should be taken seriously and that there is a need for its monitoring with regard to safety in the workplace. The established permissible exposure limit (PEL) value determined by the US Occupational Safety and Health Administration (OSHA) is 20 ppm H_2_S, specified as a time-weighted average exposure limit of a working day of 8 h [2]. During this period, the peak limit of 50 ppm H_2_S must not be exceeded [2]. Instrumental analysis methods for the detection of H_2_S include electrochemistry, spectroscopy, and gas chromatography. Probably the most commonly used method to protect employees is electrochemical sensors. These sensors show a good sensitivity to H_2_S, but they have a limited lifetime, which makes them quite expensive.

Colorimetric sensors offer a good low-cost opportunity for fast and sensitive detection of H_2_S. They are easy to handle and can be used similar to a pH-indicator strip, for example, if the color change is in the visible range. Especially in danger areas, risk is often underestimated, and the effect on the health of the people exposed often becomes visible only after many years. Furthermore, colorimetric sensors have experienced a strong growth in the analytical chemistry in the last several years.

For the detection of H_2_S, various metal complexes based on copper or iron salts are eligible. Historically, existing commercial colorimetric detection methods are based on lead acetate-impregnated paper strips. These test strips change their color from white to black/dark brown based on a reaction of Pb^2+^ with H_2_S to PbS [3]. These test papers have been classified as harmful to health and the environment, and therefore, from an ecological point of view, their use is questionable. [4]. Furthermore, they are very sensitive to oxidation by air oxygen, and light exposure leads to the fading of the strips in a relatively short time [5]. A more reliable and frequently mentioned indicator in the literature is *N*,*N*-Dimethyl-*p*-phenylenediamine. Using iron(III) ions as the catalyst, the indicator reacts through contact with H_2_S to methylene blue, which becomes visible as an intense blue color [6]. However, this indicator is also characterized by its high toxicity.

In our approach, a printable disposable sensor for monitoring H_2_S is developed using a copper(II) complex of the azo dye 1-(2-pyridylazo)-2-naphtol (H-PAN) as the color-changing material. The azo dye H-PAN has the ability to form insoluble chelate complexes with many metal ions and is commonly used as a colorimetric reagent for the quantitative determination of metal ions [7]. If H_2_S is present, the complex changes color from purple to yellow due to a reaction with S^2−^. For the deposition of a gas sensitive complex layer, a screen-printing paste was developed, and prints were performed using polyethylene (PE) foil and coated paper as the substrate. To protect the sensor against environmental influences and aging, it should be sealed subsequent to the printing process (e.g., aluminum-laminated). In this work, the color change was evaluated using a UV/Vis spectrometer with a measurement setup for diffuse reflectance. Furthermore, the fast and selective color change of the sensor due to contact with the target gas can be evaluated with the help of a camera (e.g., smartphone app) or even the naked eye.

## 2. Materials and Methods

### 2.1. Synthesis and Characterization of the Cu-PAN Complex

**Scheme 1:**
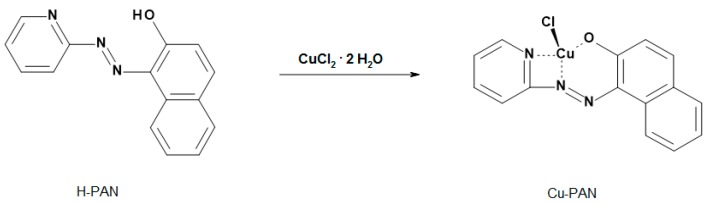
Synthesis of the Cu-PAN complex from the educts of azo dye 1-(2-pyridylazo)-2-naphtol (H-PAN) and copper(II) chloride.

The Cu-PAN complex was prepared as described in the literature [8,9] according to Scheme 1. A solution of 0.5 mmol copper(II) chloride dihydrate (≥99.99% trace metals basis, ALDRICH Chemistry, St. Louis, MO, USA) in 3 mL of distilled water was added to a solution of 0.5 mmol 1-(2-pyridylazo)-2-naphtol (H-PAN) (indicator grade, SIGMA-ALDRICH, St. Louis, MO, USA) in 75 mL of ethanol (HPLC gradient grade, ROTH, Karlsruhe, Germany). Stirring the solution at room temperature overnight resulted in a black/dark red powder, which was subsequently filtered and washed 3 times with 3 mL of ethanol and dried at room temperature. During the synthesis, the both educts of copper(II) chloride and the azo dye H-PAN formed a chelate complex and were linked by coordinative bonds [10].

The IR spectra of the synthesized complex was measured with a Bruker alpha-T FTIR spectrometer (Bruker, Billerica, MA, USA). For this purpose, 10 mg of the sample were dry-triturated with 100 mg of potassium bromide and molded into a compact at a pressure of 10 t (Perkin-Elmer Hydraulic Press, Perkin-Elmer, Waltham, MA, USA). The measurement was carried out at room temperature. IR bands: ṽ (KBr, cm^−1^) = 3053 (w), 1605 (s), 1587 (s), 1561 (w), 1547 (s), 1509 (s), 1465 (s), 1446 (w), 1412 (s), 1364 (w), 1316 (w), 1297 (w), 1249 (w), 1230 (s), 1204 (s), 1178 (w), 1148 (w), 1113 (w), 1093 (w), 1053 (vw), 1016 (w), 968 (w), 953 (w), 912 (w), 894 (w), 859 (s), 824 (s), 780 (s), 757 (s), 735 (w), 681 (vw), 664 (w), 647 (s), 582 (w), 571 (w), 557 (vw), 517 (w), 506 (w), 450 (s), 418 (w).

The analysis of the simultaneous thermal analysis (STA) measurement data (NETZSCH STA 409 C/CD, Netzsch, Selb, Germany) exhibited a high thermal stability (~90 °C) of the synthesized Cu-PAN complex.

### 2.2. Sensing Mechanism of the Cu-PAN Complex

**Scheme 2:**
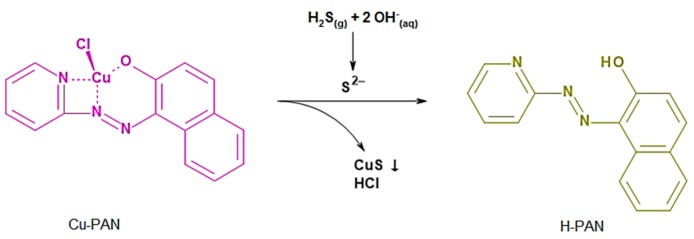
Possible sensing mechanism of the Cu-PAN complex according to Carpenter et al. [8]: Reaction of gaseous H_2_S with OH^−^-Ions to S^2−^, leading to a reaction of S^2−^ with the Cu-PAN complex, resulting in CuS and free yellow azo dye H-PAN.

Carpenter et al. described a possible sensing mechanism for the reaction of Cu-PAN and H_2_S (Scheme 2). H_2_S reacts with OH^−^_(aq)_ to S^2−^, leading to a reaction of S^2−^ with the Cu-PAN complex, resulting in CuS and free yellow azo dye H-PAN, which is perceptible to the naked eye as a color change from purple to yellow.

The reaction with H_2_S is reversible. If H_2_S is no longer present, the purple colored Cu-PAN is formed again by the reaction of the chelating ligand H-PAN with CuS. This might be due to the chelate effect, which is the enhanced affinity of the chelating ligands for a metal ion compared with the affinity of a collection of similar nonchelating ligands for the same metal.

### 2.3. Preparation of Cu-PAN on Silica

For the investigation of the sensing properties of the synthesized Cu-PAN complex, first, measurements were performed on colored silica powder. For the preparation of the sample, 1.8 mg of the Cu-PAN complex were dissolved in a 2 mL mixture of ethanol and distilled water with a ratio of 1:1 and dripped to 400 mg silica powder (99.8%, silicic anhydride, silicon dioxide amorphous, ALDRICH Chemistry, St. Louis, MO, USA). To obtain the purple-colored silica powder, the mixture was dried for 24 h at room temperature. The resulting powder agglomerates were finely grounded before the gas measurement.

### 2.4. Preparation of the Cu-PAN Test Strips

To yield a paste suitable for screen-printing, the copper(II) complex was first finely crushed and then embedded in a plasticized ethyl cellulose matrix using ethanol as the solvent. All the prints were performed on a Thieme LAB 1000 (THIEME GmbH & Co. KG, Teningen, Germany) screen-printing machine using a 180–27 polyester screen for a low level of paste application. A transparent PE foil (Autostat CT3, Gauge 75 µm, MacDermid Autotype Ltd., Wantage, UK) and a high-quality offset paper (UPM Finesse Premium Silk, 150 g/m^2^, UPM Communication Paper, Augsburg, Germany) served as the substrates. All the samples were dried at room temperature for 12 h. The layer thickness of the twofold wet-in-wet prints were determined on the PE foil to 0.9–1.1 µm by 3D laser scanning microscopy (KEYENCE VK 9700 K, Keyence Deutschland GmbH, Neu-Isenburg, Germany).

### 2.5. H_2_S Gas Measurements

**Figure 1:**
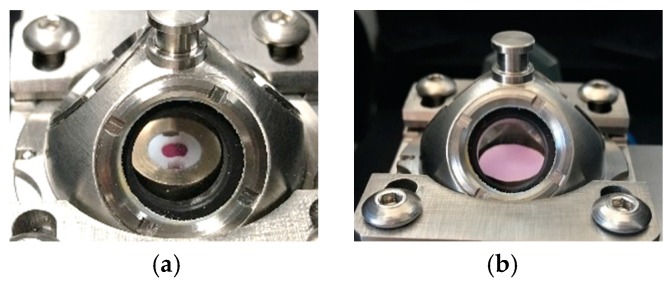
Pictures of the Cu-PAN samples in the gas measurement chamber dome: (**a**) Colored silica powder before gas exposure. (**b**) Cu-PAN layer printed on coated paper before gas exposure.

For the characterization of the colored silica powder and to assess the quality of the printed Cu-PAN layers, measurements were performed at the gas measurement station of Fraunhofer IPM. The general description of the gas measurement station is given in [11]. The color change was measured by UV/Vis spectroscopy (Perkin-Elmer Lambda900, Perkin-Elmer, Waltham, MA, USA) using a diffuse reflection accessory (Praying Mantis, Harrick Scientific Products Inc., Pleasantville, NY, USA). This measurement setup allowed for the determination of the diffuse reflectance of the solids with a high surface roughness, as well as the powder samples. To adjust the size of the prints to the measurement set-up, circles with a diameter of 10 mm were cut out of the printed indicator strips. For the measurement of the transparent PE foil, a blank paper underneath the sample served as support. The gas measurements were performed in synthetic air and a relative humidity of 40% at room temperature. Figure 1 shows the samples (silica and coated paper) in the gas measurement chamber before exposure.

## 3. Results and Discussion

### 3.1. Cu-PAN on Silica

**Figure 2:**
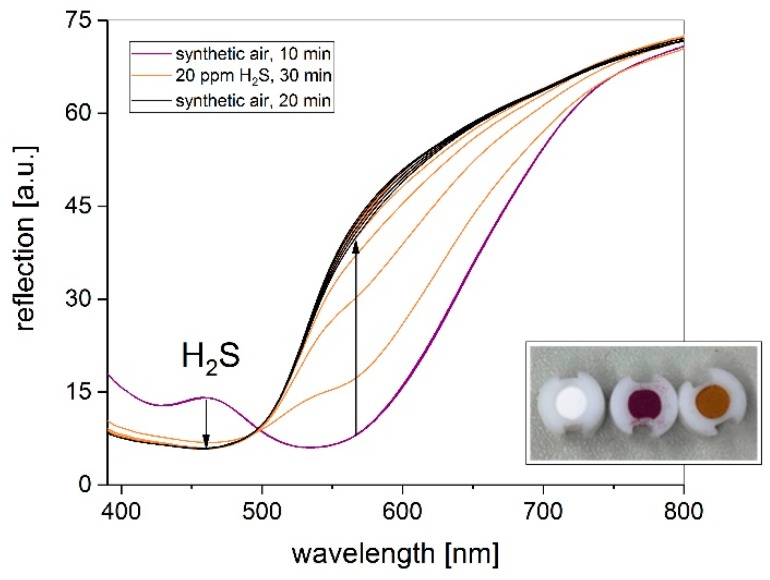
Reflection spectra of the powder sample exposed to 20 ppm H_2_S in synthetic air and a relative humidity of 40% at room temperature. Recording of 15 measurement cycles at intervals of 4 min.

Figure 2 shows the reflection spectra of the colored silica powder exposed to 20 ppm H_2_S for 30 min. Before gas exposure, the gas measurement chamber was flushed with synthetic air for 10 min and afterwards for 20 min. The measurement consists of 15 spectra, recorded in the visible wavelength range between 400 and 800 nm with a time interval of 4 min. Contact with the target gas H_2_S led to a decrease in the reflection at a wavelength range of ~400–500 nm and to an increase at ~500–750 nm, visible as a color change from purple to yellow/orange.

**Figure 3:**
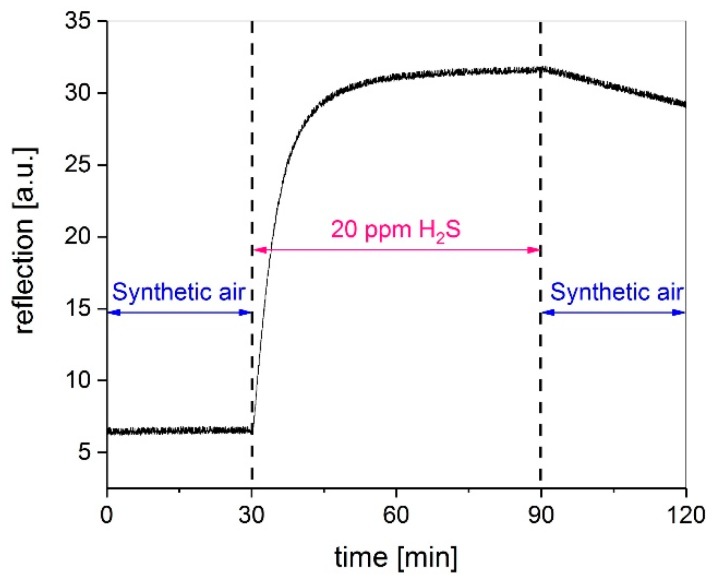
Reflection measurement of the Cu-PAN complex on silica powder at 550 nm over the measurement period. Before and after gas exposure of H_2_S for 60 min, the gas measurement chamber was flushed with synthetic air for 30 min, respectively. The measurement was performed in synthetic air at a relative humidity of 40% at room temperature.

Figure 3 shows the reflection change of the colored silica powder, reacting to 20 ppm H_2_S measured at a wavelength of 550 nm over the measurement period. The target gas was exposed for 60 min, before and after admission the reflection was recorded in synthetic air for 30 min, respectively. A T_90_ <10 min for the colored silica exposed to 20 ppm H_2_S was determined. Furthermore, after gas exposure, a slight back reaction could be observed.

### 3.2. Influence on Printing Substrate

**Figure 4:**
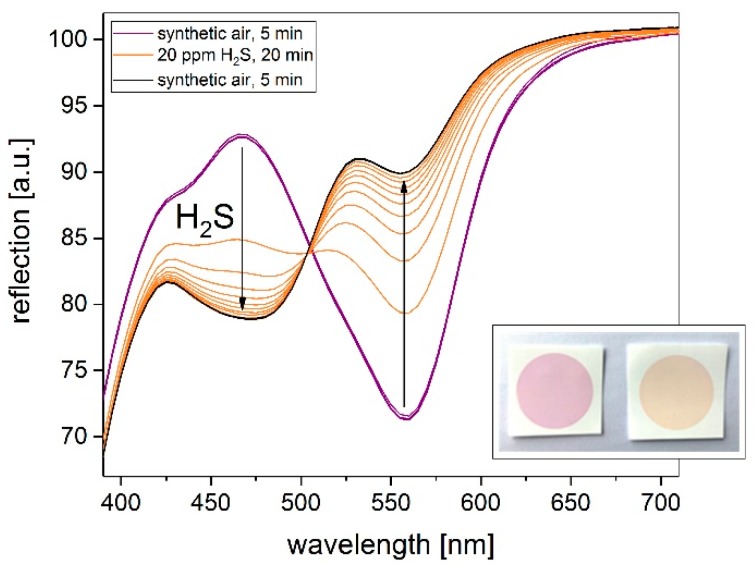
Reflection spectra of the Cu-PAN layer on coated paper reacting to 20 ppm H_2_S in synthetic air and a relative humidity of 40% at room temperature. Recording of 15 measurement cycles with a time lag of 2 min, respectively.

**Figure 5:**
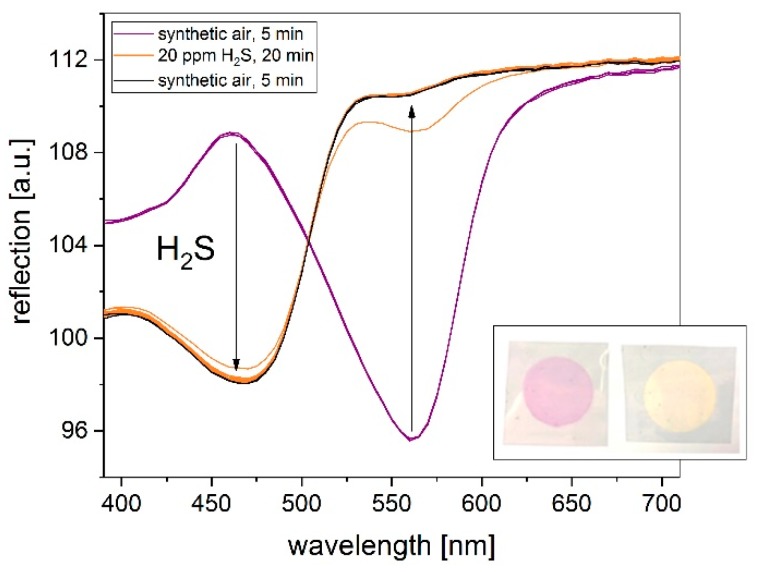
Reflection spectra of the Cu-PAN layer on polyethylene (PE) foil reacting to 20 ppm H_2_S in synthetic air and a relative humidity of 40% at room temperature. Recording of 15 measurement cycles with a time lag of 2 min, respectively.

Figure 4 and Figure 5 show the gasochromic reaction from purple to yellow of the printed Cu-PAN complex layers after H_2_S exposure (20 ppm) for 20 min on coated paper and PE foil, respectively. The measurements consist of 15 spectra, recorded with a time lag of 2 min. At the beginning and at the end of the measurement, the gas chamber was flushed with synthetic air for 5 min. The main change in reflection due to H_2_S exposition is located in the wavelength range from 460 nm (blue) to 560 nm (yellow) for both substrates. The comparison illustrates a significantly faster reaction when using PE foil as the substrate. Within less than 1 min of H_2_S exposure, the color of the layer changes from purple to yellow. The slower color change of the paper substrate might be attributed to its absorbency and the resulting larger diffusion path. However, the reflection change at the characteristic wavelength range of 460–560 nm due to the gas exposure is much higher for the coated paper.

### 3.3. Influence on Gas Concentration

**Figure 6:**
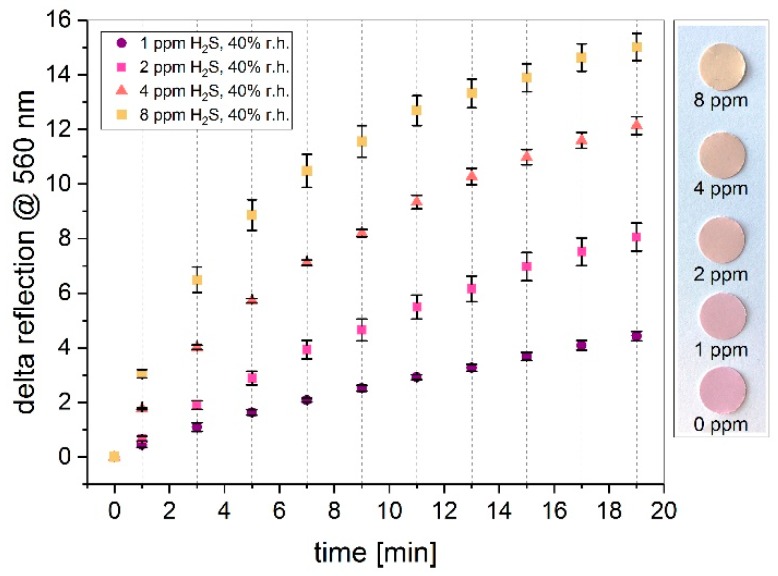
Delta reflection of the Cu-PAN layers printed on coated paper at a wavelength of 560 nm recorded over the measurement period. The samples were exposed to 1, 2, 4, and 8 ppm H_2_S for 20 min in synthetic air and a relative humidity of 40%.

**Figure 7:**
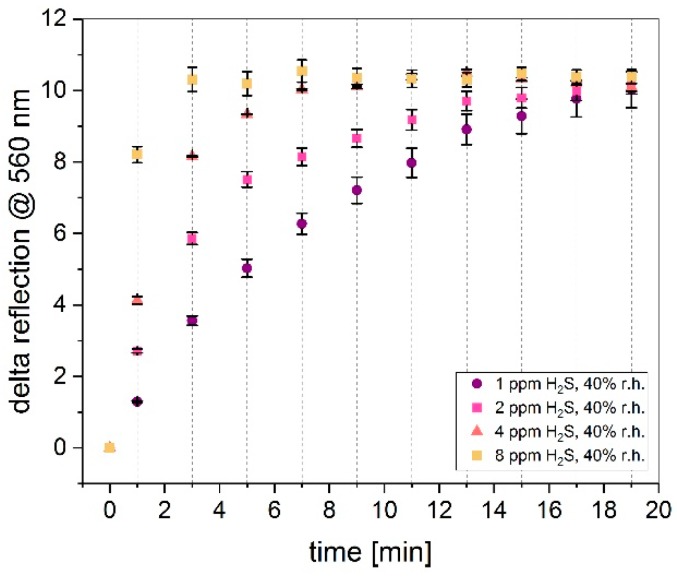
Delta reflection of the Cu-PAN layers printed on PE foil at a wavelength of 560 nm recorded over the measurement period. The samples were exposed to 1, 2, 4, and 8 ppm H_2_S for 20 min in synthetic air and a relative humidity of 40%.

A further experiment was done to investigate the dependency of the gas concentration on the speed of the color change of the printed Cu-PAN layers on the coated paper and the PE foil, respectively. A total of four printed samples of each substrate were exposed to 1, 2, 4, and 8 ppm H_2_S in synthetic air and a relative humidity of 40% for 20 min (see Figure 6 and Figure 7), respectively. To evaluate the speed of the color change, the delta reflection was recorded every 2 min during gas exposure at a wavelength of 560 nm (*n* = 3). An evaluation of the measurement results shows an increasing delta reflection with the increasing gas concentration. The usage of coated paper led to different color values at different gas concentrations. After the gas exposure, a complete color change had not occurred for any of the samples. However, when PE foil was used, a complete color change had already taken place before the end of the gas admission for all the gas concentrations.

### 3.4. Cross-Sensitivities

**Figure 8:**
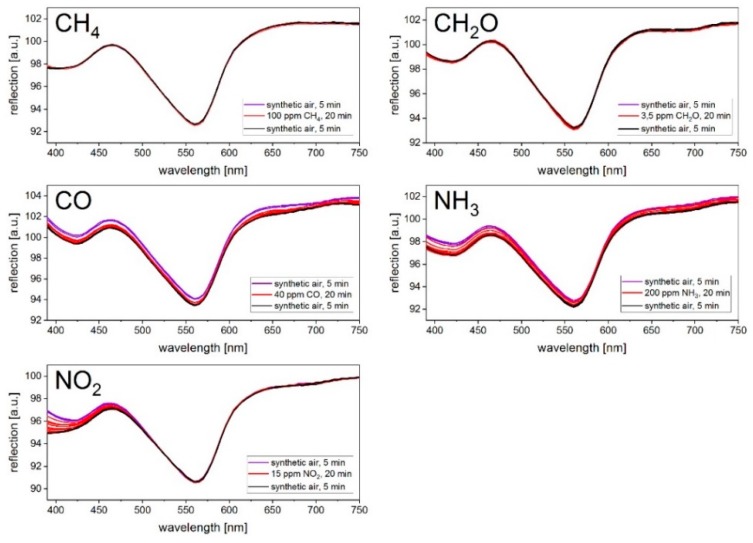
Cross-sensitivities of the Cu-PAN complex on PE foil to 100 ppm CH_4_, 3.5 ppm CH_2_O, 40 ppm CO, and 15 ppm NO_2_ in synthetic air and a relative humidity of 40% at room temperature. Recording of 15 measurement cycles with a time lag of 2 min, respectively.

To evaluate the selectivity of the printed sensors, measurements were performed for several other gases. To this end, Cu-PAN on PE foil was exposed to 100 ppm CH_4_, 3.5 ppm CH_2_O, 40 ppm CO, 200 ppm NH_3_, and 15 ppm NO_2_ in synthetic air and a relative humidity of 40% (see Figure 8). The spectra were recorded analogous to the previous H_2_S measurements (see Figure 4 and Figure 5) in a wavelength range between 400 to 800 nm and a gas admission of 20 min. The exposure to CH_4_ and CH_2_O caused no change of the measured spectra. However, if the samples were exposed to CO and NH_3_, there was a slight decrease in the reflection from 0.3% to 0.9% over the whole wavelength range. The influence of NO_2_ shows the highest change in the reflection spectra from 525 nm to smaller wavelength ranges, with a maximum change at 390 nm of 2.1%. The change due to the interfering gases was not visible to the naked eye and led to a negligible change at the critical wavelength range between 460 and 560 nm. However, measurements of the target gas H_2_S under the influence of NO_2_ led to a decrease in the reflection spectra. For the other gases, a decrease of the H_2_S sensitivity could not be determined.

### 3.5. Long-Term Stability

**Figure 9:**
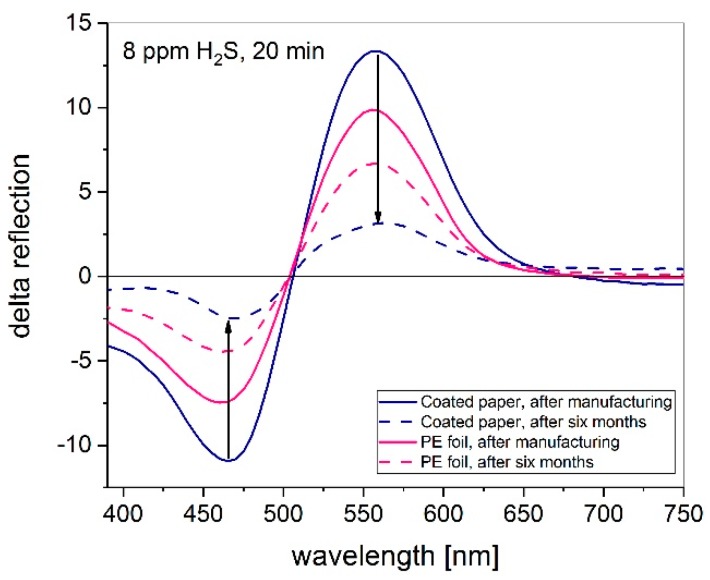
Delta reflection of Cu-PAN to 8 ppm H_2_S on coated paper and PE foil after manufacturing and after storage in ambient air at room temperature for 6 months.

For the analysis of the long-term stability, the printed samples were used and evaluated immediately after production and compared with the results after storing the substrates for 6 months in an unsealed transparent cover at ambient air and room temperature. Figure 9 shows the decrease of the delta reflection due to aging for the PE foil and the coated paper after exposure to 8 ppm H_2_S for 20 min. In the case of the PE foil, the delta reflection at the significant wavelength range of 560 nm decreased by 31.7%. For the coated paper, this value was significant higher at 76.3%. To ensure a consistent measurement result of the disposable sensor, a suitable packaging method should be developed, consequently.

## 4. Conclusions

Our investigations illustrate the ability of a printed gasochromic Cu-PAN complex layer to monitor H_2_S in ambient air, identifying the gas based on a color change from purple to yellow. The speed of the color change is influenced by the choice of the substrate. For the manufacturing of the sensor, a paste was developed, which is suitable for screen printing. With regard to the easy and inexpensive preparation of the sensor, the usage of the Cu-PAN complex could be conceivable in many environmental, biological, and industrial applications.

Based on these considerations and due to the fact that this Cu-PAN complex has a slow back reaction (complete back reaction of the printed sensors within days), it seems to be a promising material for a fast, single-use H_2_S indicator. The color change could be detected by a camera or even by the naked human eye. This research offers the opportunity to develop a low-cost, fast, H_2_S-sensitive sensor based on Cu-PAN with no need for complex and expensive components or electronics.

## References

[1] Hydrogen Sulfide The MAK-Collection for Occupational Health and Safety. https://onlinelibrary.wiley.com/doi/full/10.1002/3527600418.mb778306d0043.

[2] OSHA Standards, Occupational Safety and Health Administration Health Hazards of Hydrogen Sulfide. https://www.osha.gov/SLTC/hydrogensulfide/hazards.html.

[3] Liptàk B.G. (2003). Instrument Engineer’s Handbook, Volume One: Process Measurement and Analysis.

[4] Safety Data Sheet, Lead(II) Acetate, Carl Roth. https://www.carlroth.com/downloads/sdb/en/2/SDB_2559_MT_EN.pdf.

[5] Sanderson H.P., Thomas R., Katz M. (2012). Limitations of the Lead Acetate Impregnated Paper Tape Method for Hydrogen Sulfide. J. Air Pollut. Control Assoc..

[6] Pla-Tolós J., Moliner-Martínez Y., Verdú-Andés J., Casanova-Chafer J., Molins-Legua C., Campíns-Falcó P. (2016). New optical paper sensor for in situ measurement of hydrogen sulphide in waters and atmospheres. Talanta.

[7] Ueno K., Imamura T., Cheng K.L. (1992). Handbook of Organic Analytical Reagents.

[8] Carpenter T.S., Rosolina S.M., Xue Z.-L. (2017). Quantitative, colorimetric paper probe for hydrogen sulfide gas. Sens. Actuators B Chem..

[9] Ariza-Avidad M., Agudo-Acemel M., Salinas-Castillo A., Capitán-Vallvey L.F. (2014). Inkjet-printed disposable metal complexing indicator-displacement assay for sulphide determination in water. Spectrochimica.

[10] Köhler M., Fritzsche W. (2007). Nanotechnology: An Introduction to Nanostructuring Techniques.

[11] Kneer J., Eberhardt A., Walden P., Ortiz Pérez A., Wöllenstein J., Palzer S. (2014). Apparatus to characterize gas sensor response under real-world conditions in the lab. Rev. Sci. Instrum..

